# Software-Automated Implant Detection for Intraoperative 3D Imaging—First Clinical Evaluation on 214 Data Sets

**DOI:** 10.1007/s10278-022-00588-w

**Published:** 2022-02-10

**Authors:** Nils Beisemann, Eric Mandelka, Jan S. El Barbari, Björn Kreher, Sven Y. Vetter, Paul Alfred Grützner, Jochen Franke

**Affiliations:** 1 Medical Imaging and Navigation in Trauma and Orthopedic Surgery (MINTOS), BG Trauma Center Ludwigshafen, Ludwig-Guttmann-Str. 13, 67071 Ludwigshafen, Germany; 2 Siemens Healthcare, Erlangen, Germany

**Keywords:** Intraoperative 3D imaging, Cone-beam CT, Screw detection, Screw malposition

## Abstract

Previous studies have demonstrated a frequent occurrence of screw/K-wire malpositioning during surgical fracture treatment under 2D fluoroscopy and a correspondingly high revision rate as a result of using intraoperative 3D imaging. In order to facilitate and accelerate the diagnosis of implant malpositioning in 3D data sets, this study investigates two versions of an implant detection software for mobile 3D C-arms in terms of their detection performance based on comparison with manual evaluation. The 3D data sets of patients who had received surgical fracture treatment at five anatomical regions were extracted from the research database. First, manual evaluation of the data sets was performed, and the number of implanted implants was assessed. For 25 data sets, the time required by four investigators to adjust each implant was monitored. Subsequently, the evaluation was performed using both software versions based on the following detection parameters: true-positive-rate, false-negative-rate, false-detection-rate and positive predictive value. Furthermore, the causes of false positive and false negative detected implants depending on the anatomical region were investigated. Two hundred fourteen data sets with overall 1767 implants were included. The detection parameters were significantly improved (*p*<.001) from version 1 to version 2 of the implant detection software. Automatic evaluation required an average of 4.1±0.4 s while manual evaluation was completed in 136.15±72.9 s (*p*<.001), with a statistically significant difference between experienced and inexperienced users (*p*=.005). In summary, version 2 of the implant detection software achieved significantly better results. The time saved by using the software could contribute to optimizing the intraoperative workflow.

## Introduction

In the surgical treatment of fractures, the control of reduction and implant position by means of intraoperative imaging is an essential prerequisite for the best possible outcome [1–3]. Especially in anatomically complex regions, intraoperative three-dimensional (3D) imaging using C- or O-arms provides an additional benefit over conventional two-dimensional (2D) fluoroscopy. In various studies, 3D imaging showed significantly better results in terms of reduction and implant position compared to 2D fluoroscopy, thus contributing to the improvement of clinical outcome [4–14].

The use of intraoperative 3D imaging results in an intraoperative revision rate of up to 40% depending on the anatomical region, although there was no evidence of inadequate reduction or implant malposition in previous 2D fluoroscopy [7, 9, 11, 15]. In the event that no intraoperative 3D control is performed, findings requiring revision may only become apparent in postoperative computed tomography. This leads either to acceptance of the suboptimal findings with the risk of a worse functional outcome or alternatively to revision surgery with a potentially increased complication rate [16]. As both should be avoided, intraoperative 3D imaging is becoming increasingly established [17, 18].

However, there are also limitations to intraoperative 3D imaging. These limitations are primarily due to patient-related factors such as obesity or the patient’s positioning, which can limit the rotation of the C-arm [19]. Furthermore, intraoperative 3D imaging is susceptible to artefacts caused by the osteosynthesis material. These factors have been partially addressed by the introduction of 3D-capable C-arms with flat-panel detectors (FPD), which benefit from a larger field of view (FOV) as well as artefact reduction technology [20, 21].

The additional intraoperative time required for 3D imaging must also be taken into account. This significantly increases the duration of the operation and is mainly influenced by the time required by the surgeon for manual image evaluation [22]. The process of manual evaluation is mostly non-intuitive and particularly time-consuming, especially for users who are not trained and/or inexperienced in handling the converted 3D volume [8, 9, 15, 23, 24]. In addition to prolongation of the operating time, with its impact on complication rate for the patient and economic burden for the clinic, the manual evaluation process also involves additional risks. Settings that have to be made manually, especially by inexperienced users, tend to be not optimally adjusted which increases the susceptibility to errors and possibly the risk for missed implant malposition.

To circumvent this problem, improve the workflow of intraoperative 3D imaging and reduce the overall time required, different software applications are being developed. Those applications are designed to support the surgeon during fracture treatment by automating current manual settings for the assessment of reduction and implant position. A first step is the software-automated detection and visualization of cylindrical implants, such as screws and K-wires, in 3D data sets—the so-called *Screw Scout*®.

After clinical tests of the first version (in the following called v1) of *Screw Scout*® a second version (hereafter referred to as v2) was developed as a result of feedback given from the clinical users. Neither of the two versions has been systematically evaluated to date.

The primary study hypothesis was that the rate of correctly detected screws in v2 of the screw detection software would be significantly higher than in v1 with a concomitant lower percentage of false positives. Secondarily, it was hypothesized that using the software would save time compared to manual evaluation and that the results of v2 would be comparable to manual evaluation.

## Materials and Methods

### 3D C-arm and Software

The novel software (*Screw Scout*®, Siemens Healthineers, Forchheim, Germany) is available as a software option on the C-arm Cios Spin 3D (Siemens Healthineers, Forchheim, Germany). Cios Spin 3D features a FPD of 30 × 30 cm^2^ which creates a FOV of 16 cm^3^ (512 × 512 × 512 voxels, voxel size 0.313 mm). Furthermore, it allows motorized positioning of the isocentric gantry with rotation (0–195°), angulation (±220°) and adjustment of height (0–45 cm) [20].

The algorithm used in the software is based on the method developed by Goerres et al. The authors proposed three processing steps to automatically detect cylindrical objects such as screws and K-wires. In the first step, the image space is searched for cylindrical characteristics using orientation histograms. These characteristics are subsequently clustered according to their spatial proximity and orientation, using a density-based approach. Ultimately, these clusters as potential implants are used to initialize a cylinder-to-image registration, in the context of which localization and orientation are optimized. This step leads to the removal of false positively detected cylinders by means of an improved detection of the screw ends and the analysis of the symmetry of the image contrast perpendicular to the cylinder axis [25].

**Fig. 1:**
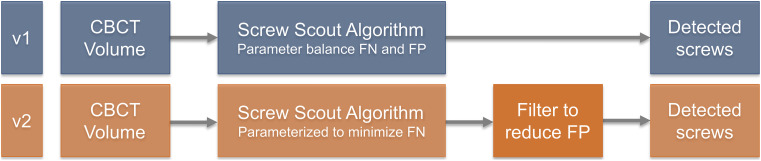
Flowchart illustrating the different approaches in v1 and v2 of the *Screw Scout*® software

During the transfer of the method into the Screw Scout® software, the different parameters of the algorithm were optimized in favor of a balance between false negative and false positive screws. This approach was used in v1 of the software investigated. v2, on the other hand, uses a different approach (Fig. 1): Here, the parameters were set in a way that minimizes the number of non-detected screws. Subsequently, a newly developed filter is used to reduce the number of false positives.

### Study Design and Data Sets

A retrospective assessment of 3D data sets of five eligible anatomic regions was performed. Data sets of wrist, spine, knee, ankle and foot were defined as eligible as intraoperative 3D imaging is most commonly used in these regions.

Data sets were extracted from the research database containing all intraoperative 3D data sets generated during surgical treatment. Only data sets acquired with Cios Spin 3D type devices between October 2019 and July 2020 were included in this study, as data sets from the period prior had been used for software development. If multiple records of a patient were available, only the intraoperative data set generated last was included in the study.

All data sets were available in high quality (110 kV, mAs/pulse based on dose control, frame rate 400/30).

In all cases, the data sets were generated on the basis of a medical indication. Since only existing image data was used for evaluation, no informed consent was required for the present study.

### Radiographic Analysis and Parameters of Detection Performance

To avoid a potential bias, manual evaluation of each data sets was performed initially, followed by the automated assessment using *Screw Scout*® version 1 and version 2.

During manual evaluation, the total number of screws and K-wires contained in each data set was acquired and defined as ground truth. The corresponding results were documented.

Subsequently, the evaluation was performed using version 1 (v1) and version 2 (v2) of the *Screw Scout*®. Various parameters were collected as displayed in the confusion matrix (Table 1).

**Table 1:** Confusion matrix with the parameters evaluated. *man eval* manual evaluation

		*Screw Scout*®	
		Implant detected	No implant detected
man eval	Existing implant	True positive (TP)	False negative (FN)
	No existing implant	False positive (FP)	--

As a result of the preceding parameters, four ratios were formed to describe the actual detection performance:

$$\mathrm{The\ true\ positive}-\mathrm{rate}\left(\mathrm{sensitivity},\ TPR=\frac{\left|TP\right|}{\left|TP\right|+\left|FN\right|}=1-FNR\right)$$

$$\mathrm{The\ false\ negative}-\mathrm{rate}\left(FNR=\frac{\left|FN\right|}{\left|TP\right|+\left|FN\right|}=1-TPR\right)$$

$$\mathrm{The\ false\ detection}-\mathrm{rate}\left(FDR=\frac{\left|FP\right|}{\left|TP\right|+\left|FP\right|}=1-PPV\right)$$

$$\mathrm{And\ the\ positive\ predictive\ value}\left(\mathrm{precision},\ PPV=\frac{\left|TP\right|}{\left|TP\right|+\left|FP\right|}=1-FDR\right)$$

The false-detection-rate instead of the false-positive-rate ($FPR= \frac{\left|FP\right|}{\left|FP\right|+\left|TN\right|}$) was used as the number of true negative implants cannot be defined. For FP implants, causes were documented. Accordingly, the location and/or cause was also identified for FN implants.

Furthermore, the time required for manual evaluation in comparison to the time required by v2 of the *Screw Scout*® software for automatic evaluation was assessed. For this part of the evaluation, five data sets of each anatomical region without false positive detections and false negative implants were randomly selected. The manual evaluation was performed by four independent researchers (two surgical residents and two experienced surgical attendings). The time needed for manual evaluation was defined as the time required for the manual adjustment of each implant contained in the data set in three planes.

### Statistical Analysis

The statistical analysis was performed using Prism 8 (GraphPad Software, Inc.) on the basis of the tabularly acquired data set using Excel (Microsoft Excel 2020, version 16.37).

Descriptive statistics are shown as frequencies and percentages for categorical variables and means and standard deviations (SD) for continuous variables. The Wilcoxon matched-pairs signed-rank test was used to analyze the central tendencies of the differences between the two software versions. The method suggested by Pratt was used for zeros and tied pairs. Paired *t*-test was used to compare time needed for evaluation by the two groups of investigators and the software. The significance level was set at *p*<0.05.

## Results

**Fig. 2:**
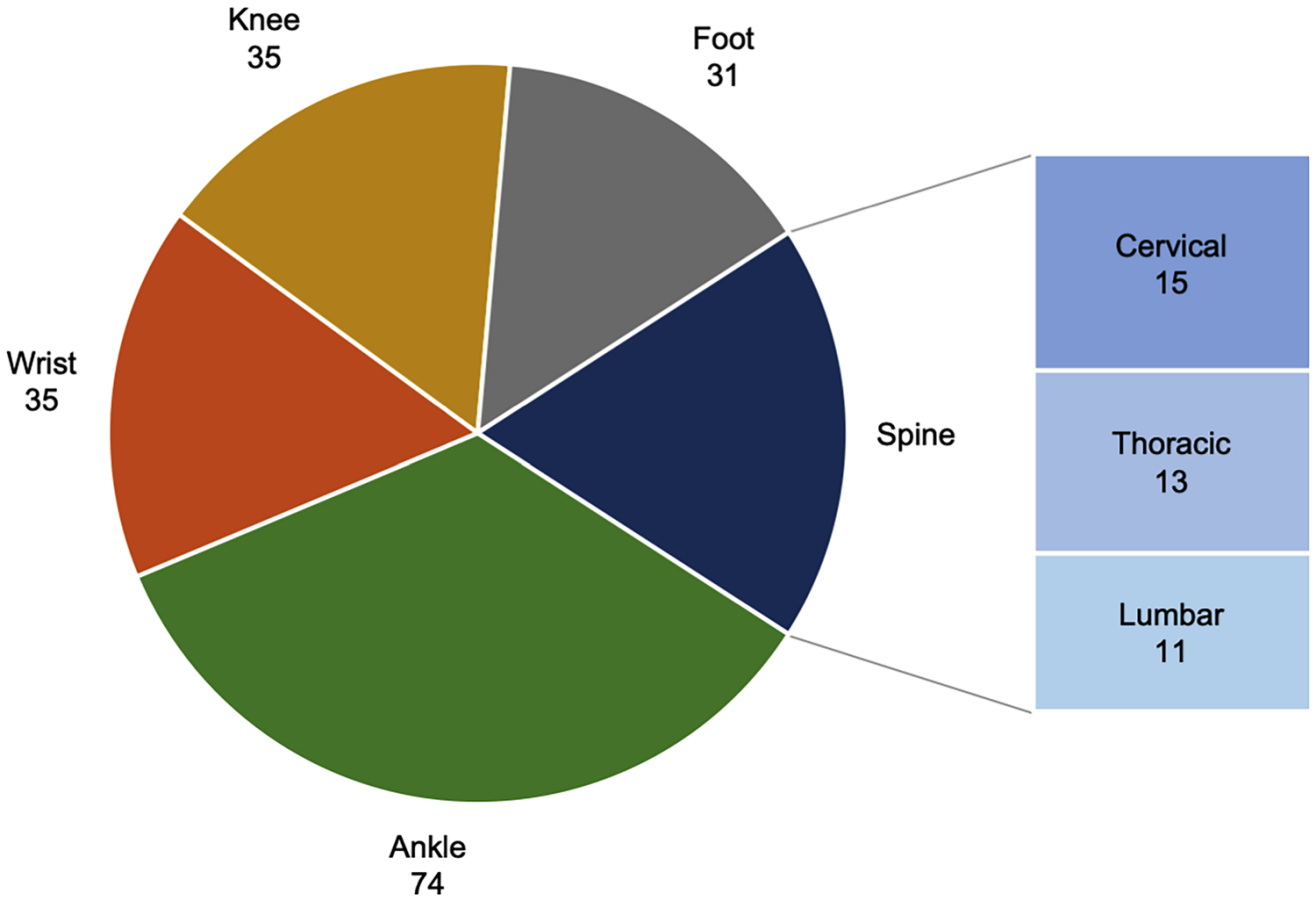
Anatomical regions and the respective number of data sets

Two hundred fourteen data sets with a total of 1767 screws and K-wires from five different anatomical regions were included. The respective number of data sets from the different anatomical regions can be read from Fig. 2.

A total of 2844 potential implants (161.0% compared to the actual implants present) were detected in v1 and 1861 implants (105.3% compared to the actual implants present) in v2.

With the detected structures actually corresponding to implants in 1514 cases (v1) and 1643 cases (v2), the overall TPR (sensitivity) was 85.7% for v1 and 93.0% for v2. Accordingly, the overall FNR was 14.3% for v1 and 7.0% for v2. Therefore, 1330 structures (v1) and 218 structures (v2), respectively, were falsely identified as implants thus reducing the FDR from 46.8 to 11.7%.

**Fig. 3:**
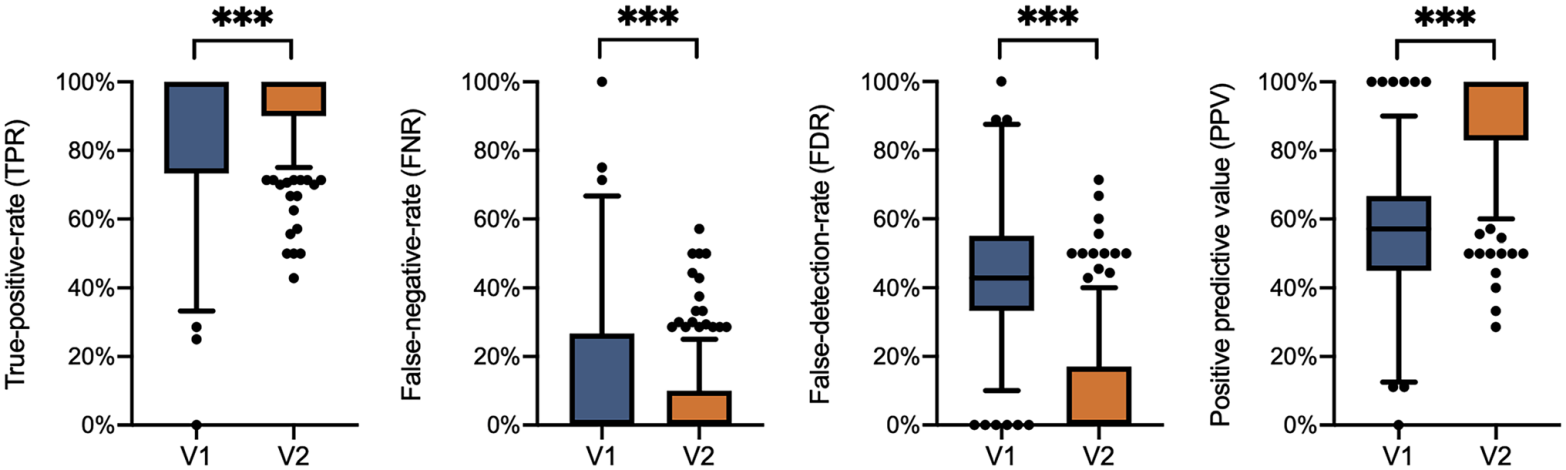
Detection performance (Whiskers displayed as 1.5×IQR as suggested by Tukey; ***p<0.001)

The overall detection performance is displayed in Fig. 3. The TPR and FDR depending on the anatomical region examined are shown in Table 2.

### False-Detection-Rate

**Fig. 4:**
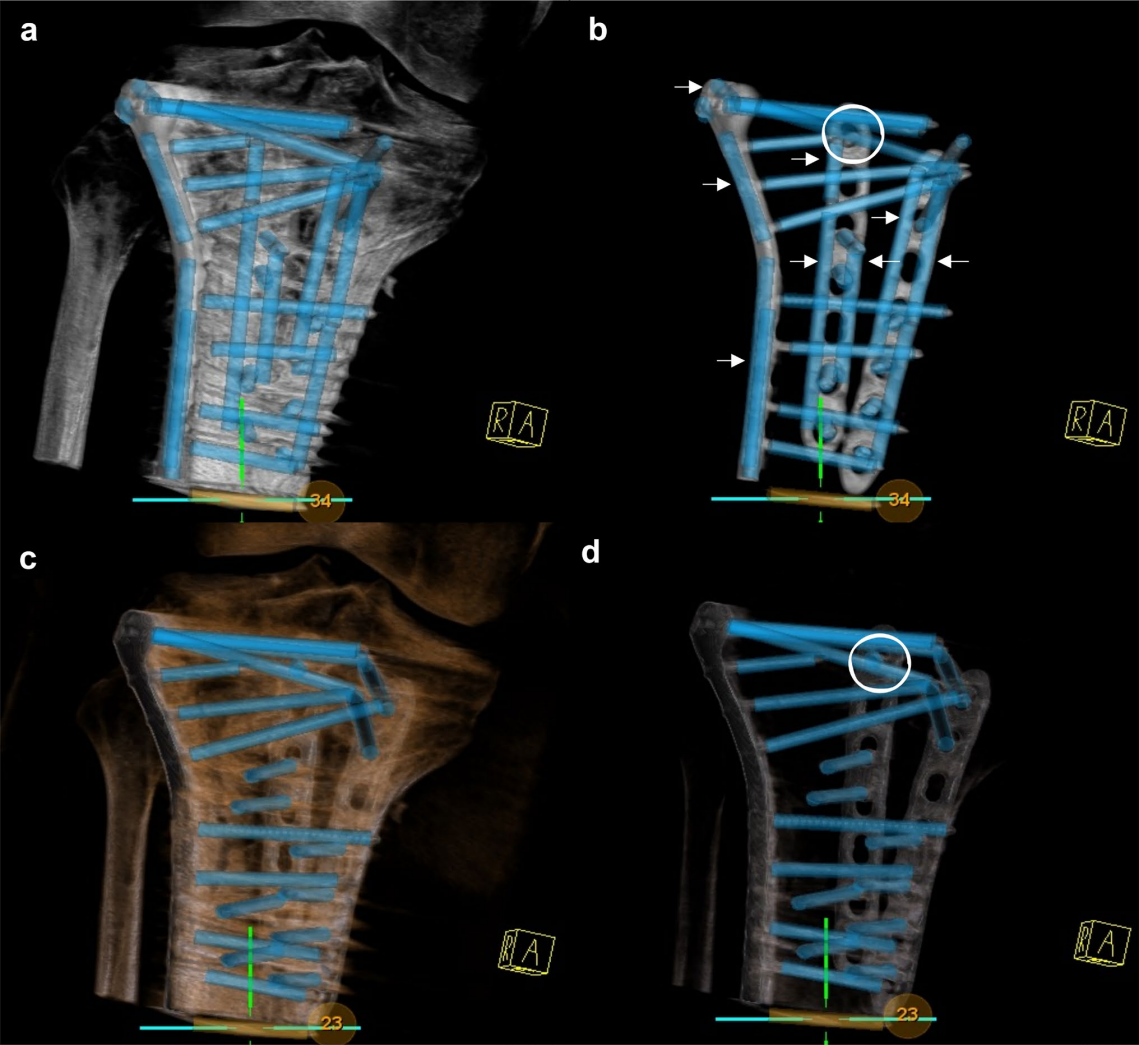
Implant detection after triple plate osteosynthesis of a tibial head fracture with 23 manually detected implants: v1 (**a**, **b**) detected 34 implants of which 22 were true positive, 12 FP implants caused by plate parts (some exemplary marked with →), one FN implant (O); v2 (**c**, **d**) detected all implants correctly, including the one which was not detected by v1 (O)

In the course of the evaluation, the causes for FP implants were investigated, as can be seen in the example of automatic implant detection after triple plate osteosynthesis of a complex tibial head fracture (Fig. 4).

Most frequently, other osteosynthesis materials such as plates as well as bone edges such as the fibular or metacarpal cortices were identified as implants by *Screw Scout*®. The FP detection of osteosynthesis materials, bones and other metals, i.e. included in central venous catheters (CVC) or endotracheal tubes (ET), was reduced by 86.1% in v2. However, in v2, more screws or K-wires were erroneously detected twice. A complete overview of the causes for FP implants is shown in Table 3.

**Table 2:** Distribution of data sets according to anatomical regions, number of manually and automatically detected implants (absolute numbers and percentage in relation to the manually detected implants) and TPR

			Manual		Version 1		Version 2	
			*n*	*(%)*	*n*	*(%)*	*n*	*(%)*
Overall (*n*=214)								
	Detected implants		1767	(100.0)	2844	(161.0)	1861	(105.3)
		True positive			1514	(85.7)	1643	(93.0)
		False detections			1330	(46.8)	218	(11.7)
Ankle (*n*=74)								
	Detected implants		655	(100.0)	848	(129.5)	613	(93.6)
		True positive			496	(75.5)	578	(88.2)
		False detections			352	(41.5)	35	(5.7)
Spine (*n*=39)								
	Detected implants		237	(100.0)	590	(248.9)	345	(145.6)
		True positive			231	(97.5)	232	(97.9)
		False detections			359	(60.8)	113	(32.8)
Wrist (*n*=35)								
	Detected implants		278	(100.0)	449	(161.5)	284	(102.2)
		True positive			214	(77.0)	255	(91.7)
		False detections			235	(52.3)	29	(10.2)
Knee (*n*=35)								
	Detected implants		344	(100.0)	573	(166.6)	352	(102.3)
		True positive			333	(96.8)	336	(97.7)
		False detections			240	(41.9)	16	(4.5)
Foot (*n*=31)								
	Detected implants		352	(100.0)	384	(151.8)	267	(105.5)
		True positive			240	(94.9)	242	(95.7)
		False detections			144	(37.5)	25	(9.4)

### False-Negative-Rate

**Fig. 5:**
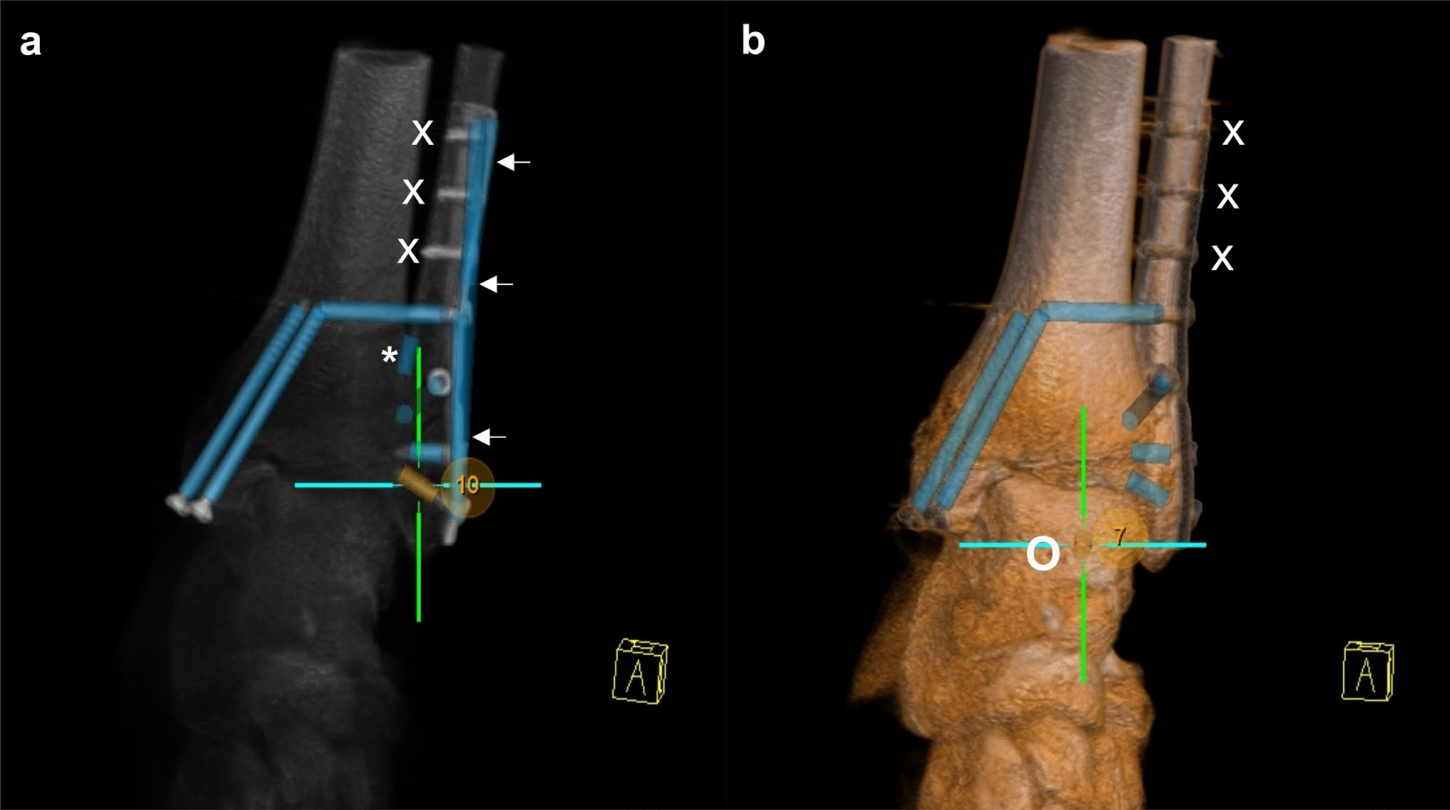
Implant detection after osteosynthesis of a bimalleolar ankle fracture (9 manually detected screws) in v1 (**a**; 10 implants detected) and v2 (**b**; 7 implants detected): (→) FP implants caused by plate, (*) FP implant caused by fibular edge, (O) FP implant caused by calcaneal bone edge, (X) FN implants of fibular osteosynthesis

The causes of FN implants were assessed specifically for the different anatomical regions (Table 4). From v1 to v2, the number of FN implants decreased to a variable extent depending on the anatomical region. The highest FNR in v2 occurred in data sets showing the wrist and the ankle. Figure 5 displays the example of implant detection after osteosynthesis of a bimalleolar ankle fracture with 9 manually detected implants. In v1, 6 implants were TP resulting in 4 FP and 3 FN implants while v2 detected 7 implants of which 6 were TP resulting in 1 FP and 3 FN implants.

**Table 3:** Causes of FP detection of screws/K-Wires with number of occurrence and FDR depending on anatomical region (*OSM* osteosynthesis materials, *DT* drainage tubes, *ET* endotracheal tubes, *EC* electric cable, *CVC* central vein catheters, *DI* dental implants)

		Version 1		Version 2	
		*n*	*(%)*	*n*	*(%)*
Overall (*n*=214)		1,330	(46.8)	218	(11.7)
	Bone	508	(38.2)	98	(45.0)
	Plate	539	(40.5)	19	(8.7)
	Implant detected twice	6	(0.5)	35	(16.1)
	OSM excl. plate (cerclage cable)	226	(17.0)	43	(19.7)
	Other (EC)	51	(3.8)	23	(10.6)
Ankle (*n*=74)		352	(41.5)	35	(5.7)
	Bone	129	(36.6)	19	(54.3)
	Plate	195	(55.4)	3	(8.6)
	Implant detected twice	0	(0.0)	4	(11.4)
	OSM excl. plate (cerclage cable)	24	(6.8)	8	(22.9)
	Other (EC)	4	(1.1)	1	(2.9)
Spine (*n*=39)		359	(60.8)	113	(32.8)
	Bone	103	(28.7)	37	(32.7)
	Plate	3	(0.8)	0	(0.0)
	Implant detected twice	4	(1.1)	19	(16.8)
	OSM excl. plate	202	(56.3)	35	(31.0)
	Other (DT, ET, EC, CVC, DI, retractors)	47	(13.1)	22	(19.5)
Wrist (*n*=35)		235	(52.3)	29	(10.2)
	Bone	136	(57.9)	25	(86.2)
	Plate	99	(42.1)	2	(6.9)
	Implant detected twice	0	(0.0)	2	(6.9)
Knee (*n*=35)		240	(41.9)	16	(4.5)
	Bone	56	(23.3)	6	(37.5)
	Plate	182	(75.8)	1	(6.3)
	Implant detected twice	2	(0.8)	9	(56.3)
Foot (*n*=31)		144	(37.5)	25	(9.4)
	Bone	84	(58.3)	11	(44.0)
	Plate	60	(41.7)	13	(52.0)
	Implant detected twice	0	(0.0)	1	(4.0)

**Table 4:** Causes of FN screws/K-wires with number of occurrence and FNR depending on anatomical region

		Version 1		Version 2	
		*n*	*(%)*	*n*	*(%)*
Ankle		159	(24.3)	77	(11.8)
	Distal fibular plate, proximal screws	117	(73.6)	61	(79.2)
	Distal fibular plate, distal screws	28	(17.6)	10	(13.0)
	Distal tibial plate, proximal screws	2	(1.3)	1	(1.3)
	Distal tibial plate, distal screws	9	(5.7)	3	(3.9)
	Screws in Volkmann’s triangle	2	(1.3)	1	(1.3)
	K-Wires	1	(0.6)	1	(1.3)
Spine		6	(2.5)	5	(2.1)
	Dorsal K-wire	3	(50.0)	2	(40.0)
	Broke-off screws	1	(16.7)	1	(20.0)
	Incorrect implant axis	2	(33.3)	2	(40.0)
Wrist		64	(23.0)	23	(8.3)
	Distal radius plate, proximal screws	47	(73.4)	11	(47.8)
	Distal radius plate, distal screws	17	(26.6)	12	(52.2)
Knee		11	(3.2)	8	(2.3)
	Tibial head plate, proximal screws	6	(54.5)	2	(25.0)
	Tibial head plate, distal screws	4	(36.4)	2	(25.0)
	K-wires	1	(9.1)	2	(25.0)
	Broke-off implants	0	(0.0)	1	(12.5)
	Implants at the edge of the field of view	0	(0.0)	1	(12.5)
Foot		13	(5.1)	11	(4.3)
	Calcaneal plate, dorsal	2	(15.4)	2	(18.2)
	Calcaneal plate, central	4	(30.8)	3	(27.2)
	Calcaneal plate, anterior	5	(38.5)	5	(45.5)
	Implants in metatarsal 2	1	(7.7)	0	(0.0)
	K-wires	1	(7.7)	1	(9.1)

### Time Needed for Evaluation

In 25 data sets, five from each of the different anatomical regions, with a total of 205 implants, screws and K-wires present in each data set were manually adjusted independently by four investigators. The two inexperienced users required an average of 147.4±76.0 s (range 33.1 to 372.1 s) per data set to do this. The two experienced users adjusted the included implants in 124.9±68.6 s on average (range 51.7 to 409.1 s). The difference between inexperienced and experienced investigators was statistically significant (*p*=0.005). The automatic detection and visualization of the implants by the software was performed in 4.1±0.4 s on average (range 3.1 to 4.9 s, *p*<0.001).

## Discussion

Besides the exact reduction of fractures, the positioning of the osteosynthesis material is a decisive factor for the functional outcome. Insufficient implant position can lead to revision surgery if it is not detected intraoperatively. In this regard, intraoperative 3D imaging offers a clear advantage over conventional 2D imaging especially in complex anatomical regions with poor assessability as shown in several studies [10, 26, 27]. The new generations of intraoperative 3D imaging devices with FPDs offer a wider field of view and better image quality, but still the manual evaluation of reduction quality and implant placement is time-consuming and susceptible to errors. Therefore, particularly for inexperienced surgeons working with 3D imaging can be challenging [25].

The aim of the study was the evaluation of a software application designed to support the surgeon using 3D imaging by automatic detection and visualization of screws and K-wires during osteosynthesis of various anatomical regions. The accuracy of two different versions of the software was investigated on 214 3D data sets of five different anatomical regions with a total of 1767 implants which had been manually evaluated beforehand. Hereby, the strengths and weaknesses of the respective versions regarding implant detection were analyzed. Furthermore, the time required for manual evaluation of the implants by two inexperienced and two experienced trauma surgeons was assessed and compared with the automatic detection and visualization.

The results show that version 2 of the *Screw Scout*® achieves significantly better results regarding the detection of screws and K-wires. This was achieved by changing the original approach of the underlying algorithm. In v1, both the minimization of the number of false positives and false negatives screws is performed in only one step, which proved to be a suboptimal compromise—as can be seen from the results presented here. In contrast, in v2, the parameters were first adjusted to detect as few FN screws as possible. Only in a second step, the FP are filtered out. As a result, the number of incorrectly detected implants—both false positive and false negative—could be substantially reduced, which is a keystone to a higher acceptance of the software amongst surgeons.

In the course of the evaluation of the false positive and false negative detected implants, different causes were identified which increasingly led to errors. The false-negative-rate was higher in regions where implants make up most of the diameter of the local bone, for example in the proximal part of the fibular plates used for treating ankle fractures. Additionally, the implantation of many implants in a comparatively small area is a common cause of incorrectly undetected implants—as, for example, in the distal plate section of fixed-angle plates used for complex distal radius fractures. Still, the false-negative-ratio in version 2 was drastically lower in the addressed problem regions than in version 1. In the other anatomical regions, where usually longer implants are implanted, the FNR could be further reduced at a low level.

The false-detection-rate was also significantly improved after adapting the algorithm in version 2 for all anatomical regions. Whereas in version 1 an average of four to five false-positive implants were detected in 10 implants, in version 2 this rate fell to one false-positive implant; a result that may be considered acceptable even after daily use in our institution.

When evaluating the cause of false positive detected implants, the software showed to be more susceptible in anatomical regions with many small bones and/or cortical bone edges. Accordingly, the false-detection-rate was higher in the distal forearm, tarsus and metatarsus due to the relatively larger diameter of the cortical bone in relation to the whole bone. By far the highest FDR was found in the spine. This is, in addition to the above-mentioned problems, due to the fact that components of the included osteosynthesis system, such as percutaneous tulips, within the field of view are often recognized as supposed implants. This may be the case because of the cylindrical shape which also applies to other sources of error such as endotracheal tubes and central venous catheters provided with metal strips when scanning the cervical and upper thoracic spine. While these sources of error obviously cannot be removed for the scanning process, the surgical team can contribute to improving the detection parameters by clearing all unnecessary metal-containing objects such as retractors and electrical cables from the area depicted.

The comparison of the time required for manual and automatic evaluation showed that the manual evaluation by both inexperienced and experienced users took on average over 2 min and in the maximum case even over 6 min—significantly longer than the automatic detection and visualization performed by *Screw Scout*®.

The significance of this study is limited by the fact that the data was evaluated retrospectively and under experimental conditions. As there is no standardized documentation of the number of screws inserted during an operation and because it cannot be ruled out that further screws were inserted following the 3D scan, the ground truth was defined after manual evaluation of the screws using 3D imaging. A reliable determination of the number of screws and K-wires present was thus possible in all cases. With regard to the manual evaluation procedure, restrictions must be made to the extent that when investigating the time required for manual evaluation, all implants contained in the data set were manually adjusted in three planes. This deviates from the standard clinical procedure to the extent that an experienced surgeon would only adjust the particularly relevant implants, for example near the articular region and/or when correct positioning cannot be reliably determined in 2D fluoroscopy. It can therefore be assumed that the actual time benefit for experienced surgeons from using the *Screw Scout*® is somewhat lower than indicated above. In comparison, the manual evaluation by the inexperienced surgeons required significantly more time compared to the experienced surgeons, although even the examiners who presented as inexperienced already had above-average prior knowledge in the handling of 3D data sets. Thus, for users completely inexperienced in working with 3D volume, the time saved is expected to be even more substantial.

As a further limitation, it must be pointed out that the regions investigated were restricted to five anatomical regions, so that, for example, no statements can be made about automatic implant detection in the pelvic region. This was done to ensure that a sufficient number of data sets could be included for each anatomical region. Since there was not an equal number of data sets for each area, an additional evaluation was performed for each anatomical region.

Once again, it should be made clear that this software is in no way intended to take over the surgeon’s responsibility for the positioning of osteosynthesis material but rather to support the surgeon in this task through automated detection and visualization, to contribute to an improved workflow in the operating room and thus to increase the quality of surgical care and overall patient safety.

Apart from a study by Goerres et al. [25], we could not identify any studies in the literature investigating the clinical use of algorithm-based implant detection and its accuracy neither on experimentally generated data sets nor on data sets obtained in the course of patient care. Although the possibility of colour highlighting implanted implants in 3D data sets from another manufacturer has been described (Titanview®, Philips Healthcare, Best, the Netherlands) [28], to our knowledge there are no scientific studies on the accuracy of the software.

The cited study from Goerres et al. also derives from our research group and was conducted during the development of the methods on which the implant detection software is based. Here, for 50 calcaneus data sets with a total of 309 implants, the true-positive-rate was reported to be 96.1% with a false-detection-rate of 2.5% which is slightly better compared with the results for this anatomical region presented above (TPR 95.7%, FDR 9.4%). In a spine data set with 50 cylindrical implants, the maximum true-positive-rate achieved by Goerres et al. was 95.0% and therefore slightly lower than the 97.9% reported here. However, the false-positive rate of 3.2% seems to be substantially lower compared to our results (32.8%). This is probably due to the fact that the data sets in the comparative study were generated in an experimental setting after completion of surgical procedures on human cadavers. The present study, on the other hand, is the first to evaluate a “ready-to-use” software-based implant detection based on data sets obtained in the course of patient care.

In the future, supported by this study, the algorithms of the *Screw Scout*® software will be further improved to achieve results that are even closer to manual evaluation. In this respect, this study was able to identify sources of error especially with regard to the different anatomical regions.

## Conclusion

The study demonstrated the significant superiority of version 2 over version 1 of the *Screw Scout*® implant detection software. With the present results, the software can be considered a useful addition to intraoperative 3D imaging to improve the surgical workflow and reduce the time needed to evaluate implant position.

## Data Availability

All data and statistics are stored in the archive of the BG Trauma Center Ludwigshafen and are available on request from the corresponding author.
